# Pupillary fluctuation amplitude before target presentation reflects short-term vigilance level in Psychomotor Vigilance Tasks

**DOI:** 10.1371/journal.pone.0256953

**Published:** 2021-09-17

**Authors:** Jumpei Yamashita, Hiroki Terashima, Makoto Yoneya, Kazushi Maruya, Hidetaka Koya, Haruo Oishi, Hiroyuki Nakamura, Takatsune Kumada

**Affiliations:** 1 Access Operations Project, NTT Access Network Service Systems Laboratories, Nippon Telegraph and Telephone Corporation, Yokosuka, Kanagawa, Japan; 2 Department of Intelligence Science and Technology, Graduate School of Informatics, Kyoto University, Kyoto, Kyoto, Japan; 3 Human Information Science Laboratory, NTT Communication Science Laboratories, Nippon Telegraph and Telephone Corporation, Atsugi, Kanagawa, Japan; Julius-Maximilians-Universität Würzburg, GERMANY

## Abstract

Our daily activities require vigilance. Therefore, it is useful to externally monitor and predict our vigilance level using a straightforward method. It is known that the vigilance level is linked to pupillary fluctuations via Locus Coeruleus and Norepinephrine (LC-NE) system. However, previous methods of estimating long-term vigilance require monitoring pupillary fluctuations at rest over a long period. We developed a method of predicting the short-term vigilance level by monitoring pupillary fluctuation for a shorter period consisting of several seconds. The LC activity also fluctuates at a timescale of seconds. Therefore, we hypothesized that the short-term vigilance level could be estimated using pupillary fluctuations in a short period and quantified their amplitude as the Micro-Pupillary Unrest Index (M-PUI). We found an intra-individual trial-by-trial positive correlation between Reaction Time (RT) reflecting the short-term vigilance level and M-PUI in the period immediately before the target onset in a Psychomotor Vigilance Task (PVT). This relationship was most evident when the fluctuation was smoothed by a Hanning window of approximately 50 to 100 ms (including cases of down-sampled data at 100 and 50 Hz), and M-PUI was calculated in the period up to one or two seconds before the target onset. These results suggest that M-PUI can monitor and predict fluctuating levels of vigilance. M-PUI is also useful for examining pupillary fluctuations in a short period for elucidating the psychophysiological mechanisms of short-term vigilance.

## Introduction

Vigilance-related task performance fluctuates in different tasks ranging from flying [1] and driving [2] to radiology [3]. Moreover, decreased vigilance could result in severe or even deadly consequences. Therefore, it is beneficial to develop techniques that effectively monitor real-time decrements in vigilance.

A potentially helpful physiological marker for estimating vigilance decrement is the pupillary response. It is known that there is a strong link between real-time pupillary responses and activation of the Locus Coeruleus (LC) and Norepinephrine (NE) system (the LC-NE system [4]). Furthermore, Oken, Salinsky, and Elsas [5] suggested that vigilance is innervated by the NE system, including the LC. Based on these, numerous attempts have been made to use the pupilar diameter as an indicator of task performance related vigilance, including driving [6], flying [7], and radiology [8]. These studies have often calculated the extent of the pupilar diameter.

Another possible approach to estimating the long-term vigilance level takes advantage of pupillary fluctuations’ characteristics. This method measures the extent to which the pupilar diameter fluctuated instead of its diameter. The stable autonomic nervous system (ANS) under vigilance results in a relatively stable pupilar diameter. In contrast, fluctuations between sympathetic and parasympathetic control under reduced long-term vigilance result in larger fluctuations of the pupilar diameter [9], possibly controlled from the LC-NE system [10], which are known as "sleepiness (or fatigue) waves." Very slow frequencies are typically characteristic of sleepiness waves. For instance, Wilhelm, Wilhelm, Lűdtke, Streicher, and Adler suggested that the Pupillary Unrest Index (PUI) calculated from pupillary fluctuation amplitude (the degree of absolute changes in pupilar diameter) in the low-frequency band is designed to capture pupillary fluctuations below 0.8 Hz [11]. An increase in the PUI is known to reflect a decline in Psychomotor Vigilance Task performance every several hours [12] (i.e., a decrease in the long-term vigilance level). Nevertheless, using the PUI to estimate participants’ vigilance levels in real-time during a task is challenging. This is because a single PUI is calculated from the time-series of pupilar diameters in the resting state for 11 minutes while interrupting the task. However, estimating real-time (e.g., trial-by-trial) vigilance levels during a task is crucial because it would facilitate identifying the viewer’s levels of vigilance for different activities.

The data for estimating the level of vigilance in real-time is the frequency of LC’s neuronal activity, including long and short timescales (from 0.1 to 20 Hz) [13, 14]. Pupilar diameter fluctuations that are conventionally observed as sleepiness-waves only capture the long timescale (below 0.8 Hz, in PUI), possibly reflecting ANS-related LC’s tonic activity from 0.1 to 5.0 Hz [13, 14]. Therefore, it takes a long time to collect reliable data on pupillary fluctuations, which leads to limitations, such as the need to isolate participants for an extended period to estimate their long-term vigilance levels. However, some LC activities occur at a high frequency on short timescales, possibly reflecting LC’s phasic activity from 10 to 20 Hz [13, 14]. Such LC activities are assumed to be related to short-term states such as engagement with a current task by enhancing task-relevant stimuli [13, 14] rather than with long-term states such as sleepiness. Because LC activities fluctuate at a high frequency, pupillary fluctuations on small timescales increase as the short-term level of vigilance may decrease on a short-term (e.g., trial-by-trial) basis, assuming that randomness among neural activities in the LC at a high frequency also increases as engagement with the task decreases [13, 15]. If pupilar diameter fluctuations reflect LC’s vigilance state in the short term, then merely measuring pupillary fluctuations during a task for a short period would enable estimating real-time vigilance levels without interrupting the task.

Therefore, we propose using the Micro-Pupillary Unrest Index (M-PUI), which is calculated from the short-term pupillary fluctuation amplitude within a short time. Wilhelm, Wilhelm, Lűdtke, Streicher, and Adler [11] described the original PUI calculation procedure. In the M-PUI, similar to the PUI, the degree of ’unrest’ in the pupilar diameter is calculated by adding absolute dilation values and pupillary fluctuation constrictions (calculating pupillary fluctuation amplitude). However, we changed the timescale of pupillary fluctuations that we captured. Frequencies of pupillary fluctuations captured by M-PUI have a sufficiently small time resolution for assessing M-PUI in the short-term. We expected that LC activity fluctuations during low vigilance periods would be reflected in larger pupilar diameter fluctuations even in the short-term on a trial-by-trial basis within each participant.

**Fig 1:**
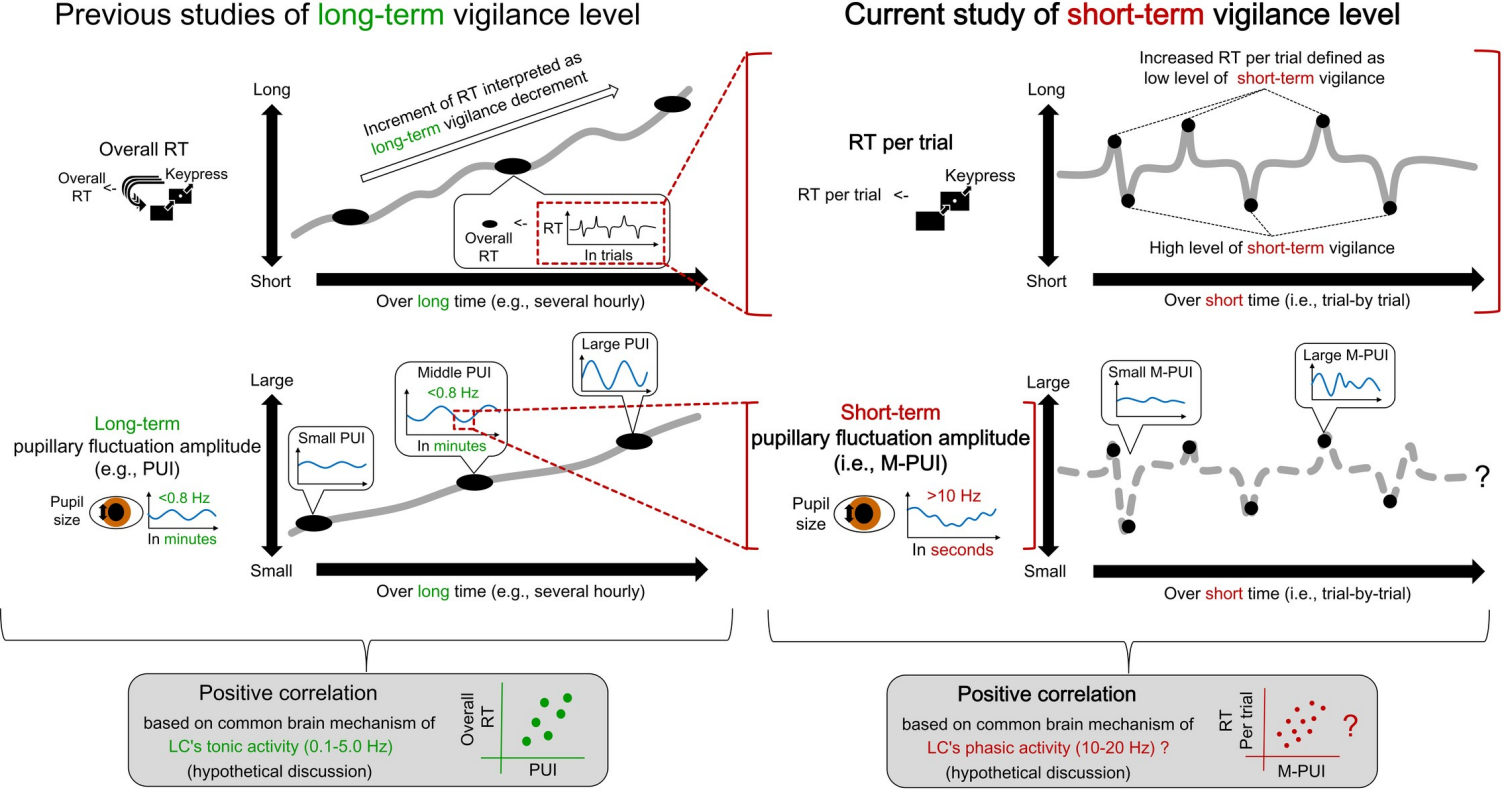
A conceptual summary of the current study. Previous studies, which correspond to the left panel, have confirmed the positive correlation between overall RTs in each vigilance task session (e.g., the numbers of long RTs in each PVT session) and long-term pupillary fluctuations amplitudes measured over several minutes immediately before each PVT session (e.g., PUIs below 0.8 Hz), which were acquired every several hours. The long-term increase in the overall RT is interpreted as a decrement in long-term vigilance. The underlying mechanism of this relationship could be ANS-related tonic activity of the LC from 0.1 to 5.0 Hz. On the other hand, the current study, which corresponds to the right panel, examined the positive correlation between the RTs per trial within one session of PVT and the short-term pupillary fluctuations amplitudes per trial within one session of PVT (i.e., M-PUIs measured with a fine temporal resolution of 10 Hz or more immediately before the target presentations during trials). In this study, the increase in RT per trial was defined as a decrease in short-term vigilance level. The underlying mechanism of this relationship might include the phasic activity of the LC from 10 to 20 Hz.

We examined the intra-individual trial-by-trial relationship between short-term vigilance levels and M-PUI calculated during a short period, just before measuring vigilance for an ongoing task. Fig 1 shows the conceptual summary of the current study. From a practical perspective, establishing this relationship was considered the first step in estimating real-time vigilance levels. Simultaneously, from a theoretical perspective, these trial-by-trial calculations were considered to reveal the relationship between the short-term LC activities at a high frequency and short-term vigilance levels. Thus the effectiveness of M-PUI in estimating the short-term levels of vigilance was examined from both practical and theoretical perspectives.

## Experiment

We examined the validity of M-PUI as an indicator of short-term vigilance levels using a simple reaction task [16] based on the Psychomotor Vigilance Task (PVT) [17]. The PVT participants were required to respond to a target presented at a variable interval by pressing a button as quickly as possible. One essential measure indicating that task performance could be interpreted as reflecting vigilance is the decline in task performance over a long time (e.g., throughout a day) [17]. Therefore, a standard procedure for measuring long-term vigilance levels is to repeat a task at intervals (e.g., for several hours) during continuous wakefulness and observing any possible decreases in task performance [5]. PVT is established as the standard task for capturing pure performance decrements over some time after excluding the practice effects of repeated trials [17]. As a result, the number of "lapses (long RTs over 500 ms)" in each PVT session increases (i.e., long-term vigilance level decreases) in each repeated session [18], along with an increase in long-term pupillary fluctuation amplitudes (i.e., PUI) [12]. Therefore, PVT is considered a vigilance task, and PUI indicates the long-term vigilance level. As noted in the Introduction, LC’s tonic activity could be the underlying mechanism of these relationships.

Short-term vigilance level fluctuation mechanisms during PVT have been investigated recently. Recent studies have used RTs per trial as an indicator of short-term vigilance levels and examined their psychophysiological correlations [19, 20], suggesting the role of LC-NE in short-term vigilance levels. For example, it has been reported that trials with shorter RTs in participants with normal sleep have been associated with greater involvement of the sustained attention networks, which are closely related to the cortical activation levels regulated by the LC-NE [19, 21]. Moreover, shorter RTs are associated with greater pupilar diameter reflecting LC-NE activation [20]. This previous study also discussed that LC’s phasic activity could explain these short-term relationships’ underlying mechanisms [20]. However, the intra-individual trial-by-trial relationship between short-term pupillary fluctuation amplitude (i.e., M-PUI) and RT has not been established (cf. Fig 1).

We also used RTs per trial as a measure of short-term vigilance levels. It was expected that an increase in M-PUI would be associated with an increase in RT on a trial-by-trial basis, probably via LC activity fluctuations, which are reflected in larger pupillary fluctuation amplitudes in a short timescale. If M-PUI reflects RT changes, we expected that these short-term pupillary fluctuations would indicate a participant’s vigilance level on a trial-by-trial basis.

### Method

We examined the extent to which the M-PUI reflects the short-term levels of vigilance by covering a wide range of cases, from simple analyses that follow practical guidelines to sophisticated analyses with theoretical implications. In these analyses, we believe that there is a trade-off between practice and theory.

From a practical perspective, it is appropriate to examine the relationship between short-term vigilance and M-PUI using a hard threshold that needs no adjustment for background factors, assuming a real-world situation in which such factors cannot be considered or controlled. The effect size of M-PUI should be large enough to show statistical significance even in such a ’practical’ situation.

On the other hand, such an analysis does not provide generalizable results in terms of theory because the effect of M-PUI and the background factors are not separated. Therefore, from a theoretical perspective, it is also necessary to examine the exact effect size of M-PUI under a ’theoretical’ setting in which the background factors are controlled.

Based on these assumptions, we considered individual differences to be background factors, and we conducted data analyses that progressed from those that do not take individual differences into account to those that do. We first examined the relationship using a hard threshold that needed no adjustment for individuals. We then used individually adjusted thresholds to produce equivalence for all individuals, thus controlling RT differences among individuals. Finally, in the most theoretical test, we examined the relationship between individually normalized M-PUIs and individually normalized RTs on all trials, without thresholding. We also conducted two analyses that provide significant insights from practical and theoretical perspectives (see details in the data analysis section; Fig 4).

#### Participants

Participants in the experiment were people with a normal or corrected-to-normal vision that applied for a part-time job (N = 20, 15 females Age range 20–43 years). They were recruited from outside the laboratory and received payment for their participation. Recruitment of participants and experimental procedures was approved by the NTT Communication Science Laboratory Research Ethics Committee (Reference Number H29-004) and was conducted according to the 1964 Declaration of Helsinki. We obtained the written informed consent from all observers in this study.

#### Apparatus and stimuli

Participants were seated 60 cm from an LCD monitor (144Hz, 27-in., 1920×1080 pixels). Stimuli were presented using Python and Psychopy2 on a black background. The target was a white dot (2.9 visual angles) in the center of the screen. The SR Research Eyelink 1000 was used to record eye movements at a sampling rate of 1,000 Hz. Data from the participants’ left eyes were used. We independently stabilized each participant’s head with a chin-rest during the entire task.

#### Procedure and design

Each participant performed eight tasks, one of which was analyzed for this study. The order of the tasks was randomized. Each task took about ten minutes and was followed by a break of ten minutes. In each session, two participants were paired, and one of them took a break while the other performed a task. The total procedure was 180–210 minutes, including task preparation.

**Fig 2:**
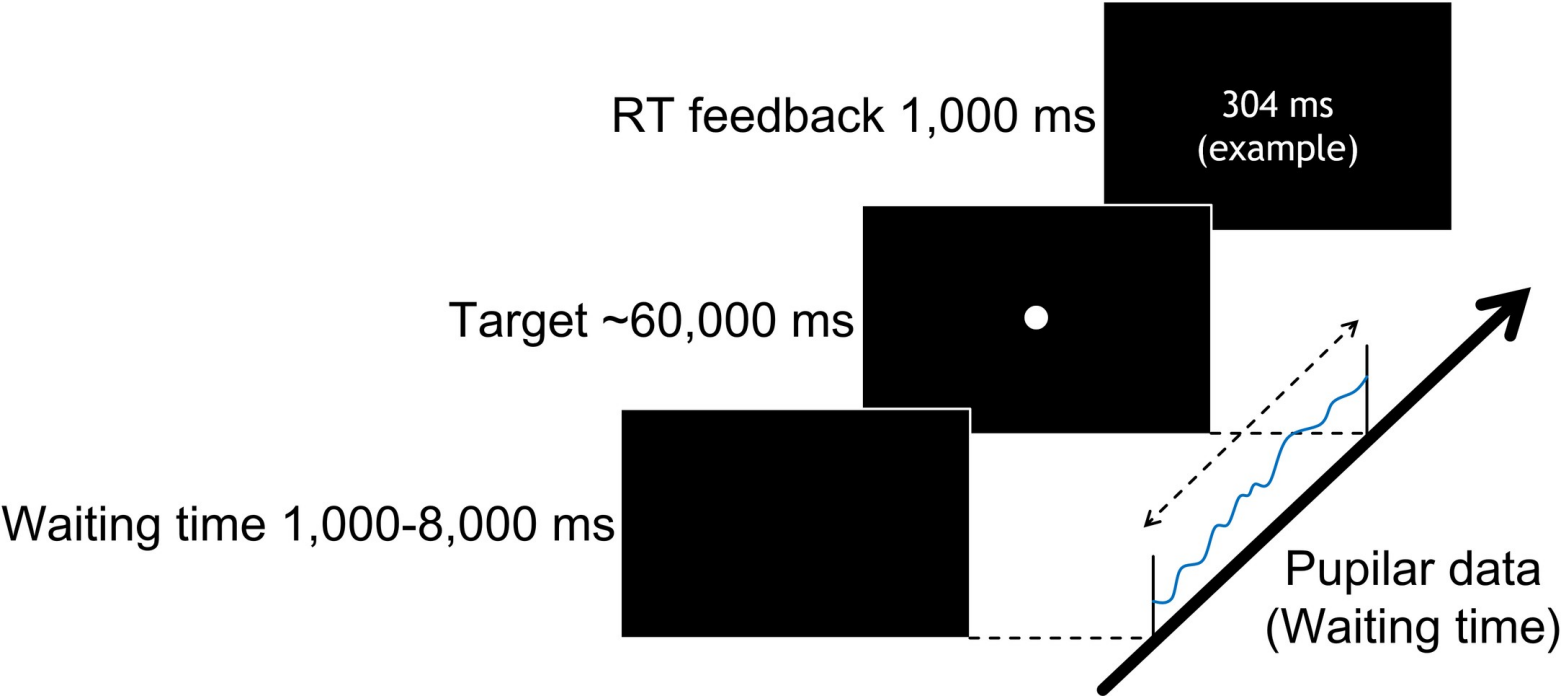
The serial flow of elements in one trial of the Psychomotor Vigilance Task. After a random interval (1,000 to 8,000 ms), a target (white dot) appeared. Participants were asked to press a key for the appearance of the target as quickly as possible. After the keypress response, the time from the appearance of the target to the keypress was displayed. The section for measuring the pupilar diameter is illustrated (from the start of the trial to the target appearance).

The PVT ran for about 10 min. After providing informed consent and calibrating the eye tracker, participants performed the PVT (Fig 2). Participants were instructed to respond to a target presented at a variable interval (equally distributed from 1,000 to 8,000 ms in 250 ms increments) by pressing the "space" key on the keyboard as quickly as possible. Participants performed four trials×29 interval conditions. In response to the "space" keypress, reaction time was displayed for 1,000 ms. If a response was made before the target’s appearance, then the message "False Alarm!" was displayed. If a response was not made within 60 seconds, the message "Miss!" was displayed. In each case, after the message disappeared, the subsequent trial restarted.

#### M-PUI calculation

The time interval from the start of the trial to the target appearance was defined as the measurement section. We excluded the time-series of the pupilar diameter when the eye was closed before the calculation. Blink detection was conducted by assuming that a blink has occurred when the pupilar diameter’s constant value fell below the threshold and continued to fall until it exceeded the threshold. The threshold was determined as the median pupilar diameter of the entire task × 0.5 to account for individual differences in the pupilar diameter baseline. The blinking time and 200 ms before and after blinks that could cause artifacts were also defined as blinks (cf. [22]).

**Fig 3:**
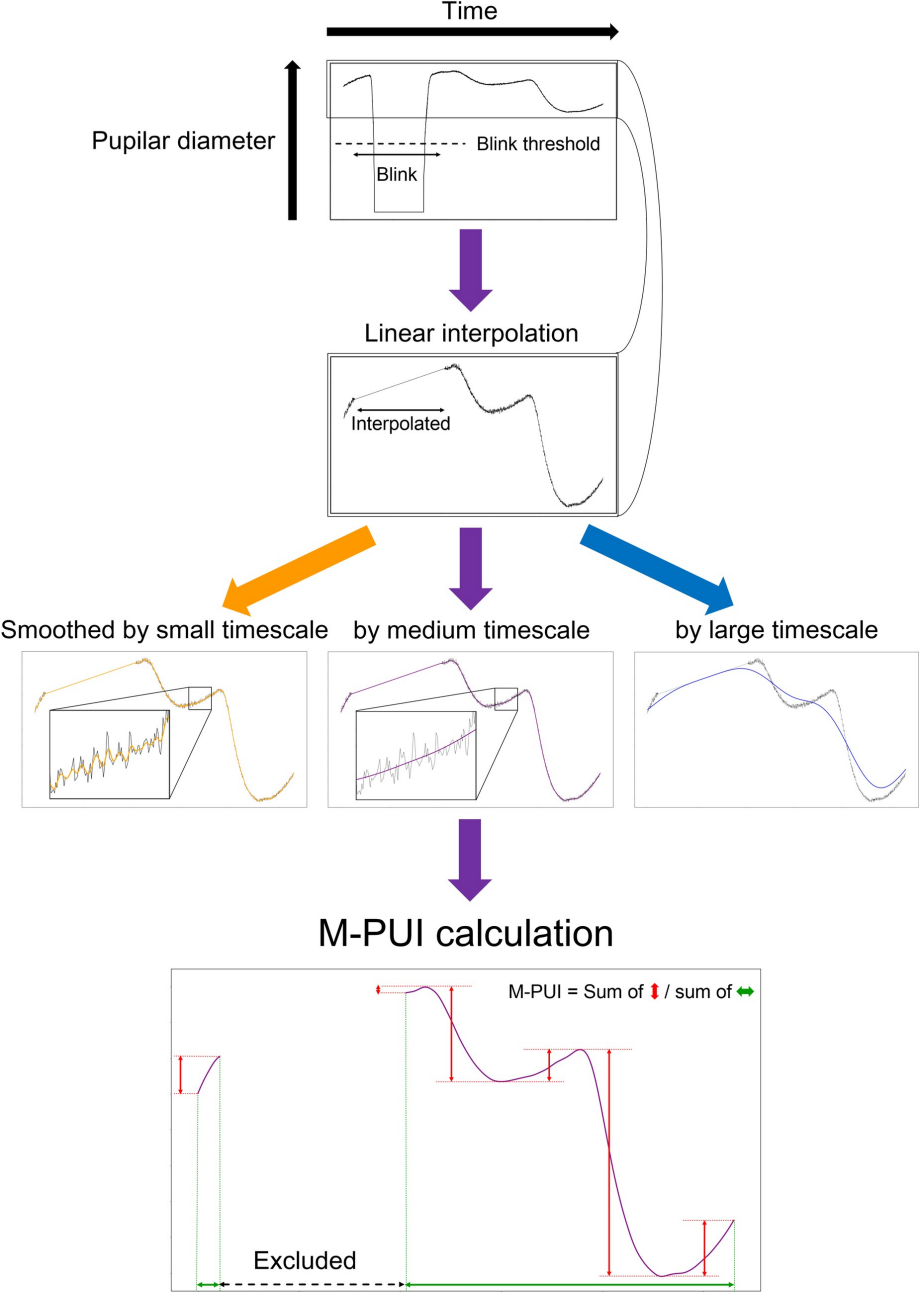
A Conceptual example of M-PUI calculation. The purple arrows in this figure represent the flow of M-PUI calculation. The black line in the first block represents the raw pupilar diameter’s time series. First, a blink was detected using the rule that a blink occurs when the pupilar diameter’s constant value falls below the threshold. Second, the pupilar diameter’s time series was linearly interpolated during the blink. Third, noisy time series with raw pupilar diameters were smoothed. Different smoothed data are obtained using differently sized time windows (orange, purple, and blue lines). Note that small fluctuations can be seen for smoothing with small timescales, but not for other cases. Fourth, we calculated M-PUI in the smoothed pupilar diameter’s time series by using a medium timescale in this example. Then, we calculated the absolute value for the degree of change (the sum of the magnitude of red arrows) in pupillary fluctuations. Finally, the absolute degree of change was divided by the length of a specific interval (the sum of the magnitude of green arrows).

As mentioned above, the M-PUI calculation procedure was inspired by Wilhelm, Wilhelm, Lűdtke, Streicher, and Adler [11], who suggested that low-pass filtered time-series of pupilar diameter should be differentiated, and the absolute values of the differentiated values should be averaged as PUI. PUI was originally designed to capture the very slow pupillary fluctuations below 0.8 Hz in the resting state, whereas M-PUI is designed to capture the degree of absolute change in pupillary fluctuations that occur in seconds. Therefore, the time-series of the pupilar diameter smoothed by different time windows, which were smaller than those used in the original PUI calculation, were differentiated, and their absolute values were averaged as M-PUI. We used the time-series of the pupilar diameter smoothed by the mountain-like distributed weights arrays (in this case, Hanning windows) of 10, 20, 30, 40, 50, 60, 70, 80, 90, 100, 120, 140, 160, 180, 200, 300, 400, 500, 600, 700, 800, 900, and 1000 ms. Smoothing was performed by calculating the moving averages while moving the Hanning windows in the time-series data of raw pupilar diameters. These procedures in which the blinks were carefully removed in two stages are shown in Fig 3. Firstly, the time-series of pupilar diameter before smoothing were linearly interpolated during the blinks to ensure stable smoothing. Secondly, the smoothed time-series of pupilar diameter during the blinks were excluded when calculating the M-PUI after smoothing.

#### Data analysis

**Fig 4:**
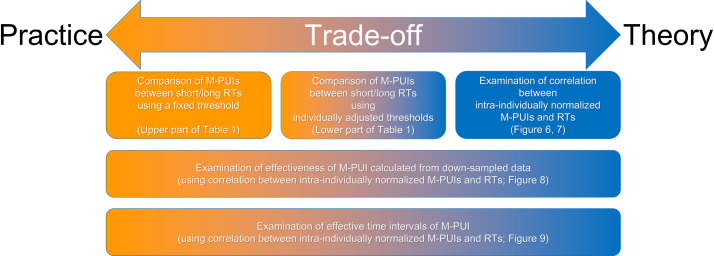
Positions of data analyses in the practice-theory trade-off.

The data analysis examined the extent to which the M-PUI reflects short-term vigilance levels by examining a wide range of cases based on the trade-off between practice and theory (Fig 4). Trials were divided into the fastest and slowest RTs using individually adjusted thresholds (i.e., percentile points) in previous studies examining physiological correlates of trial-by-trial RT variations in PVT. The physiological indicators were compared between these groups of trials [19, 20]. This analysis corresponds to the top middle panel of Fig 4. We expanded the previous analysis for both practical and theoretical purposes.

First, from a practical perspective, it is appropriate to examine the relationship between short-term vigilance and M-PUI using a hard threshold that needs no adjustment for individual differences (top left panel of Fig 4). If M-PUI reflects the short-term levels of vigilance even without taking individual differences into account, we can show the effectiveness and robustness of M-PUI in practice. This is because the mere fact that physiological indicators (i.e., M-PUIs) differ according to relative RTs within an individual does not indicate any practical effects. For example, imagine that we are trying to prevent an automobile accident, and the drivers have to react in some way within a fixed time that is common to everyone, regardless of their individual levels of overall vigilance. In such cases, it is necessary to estimate the absolute levels of short-term vigilance rather than relative vigilance. For this purpose, we first examined differences between the mean M-PUI calculated from trials with an RT over 500 ms (long RT) and the mean M-PUI calculated from trials with an RT less than or equal to 500 ms (short RT) per participant. Note that 500 ms was chosen to ensure the relevance of M-PUI to previous studies that have traditionally used the number of "lapses (of RT of more than 500 ms)" as an indicator of decreased long-term vigilance [12]. One-tailed paired-samples t-tests were used to compare the means of each group. We excluded from further analyses trials with misses, false alarms, or anticipatory responses (RT < 150 ms) and trials conducted with the eyes closed during the complete measurement phase. We also examined cases in which the threshold was reduced by 50 ms to 450 and 400 ms. If M-PUI reflected the fluctuating level of vigilance in each trial, we expected larger M-PUIs for longer RTs than for shorter RTs.

Next, following previous studies [19, 20], we divided the trials into two short-term levels of vigilance based on a threshold that would be the same for all individuals while controlling RT differences among individuals (the top middle panel of Fig 4). The adjustment of the threshold for each participant was necessary to provide results that could be generalized theoretically, although it lacked the simplicity of estimation in real-world settings. In this analysis, the RTs of each individual were sorted in order of length, and RTs longer than the 90th percentile point (long RT) were considered to be trials performed with a low short-term vigilance level, while RTs shorter than the 90th percentile point (short RT) were considered to be trials performed with a high short-term vigilance level. We then examined differences between the mean M-PUI calculated from trials with long RT and the mean M-PUI calculated from trials with short RT. We also examined cases in which the threshold was reduced by 30 percent to the 60th and the 30th point.

In the most theoretical test, we examined the relationship between intra-individually normalized M-PUIs and intra-individually normalized RTs on all trials (the top right panel of Fig 4), rather than dividing the data by the threshold. This is because previous studies’ dichotomous approach [19, 20] might not be theoretically adequate to explain the relationships between the overall RT distribution and physiological indicators such as M-PUIs. Therefore, we used correlation coefficients between intra-individually normalized M-PUIs and intra-individually normalized RTs to evaluate the "steepness" of the linear relationship between M-PUIs and RTs. Note that the two variables normalized to the zero mean and one standard deviation used in this analysis make the correlation coefficient between these two variables identical to the regression slope (i.e., "steepness" of the linear relationship). In this procedure, we first normalized RT and M-PUI within individuals (and within different smoothing sizes, in the case of M-PUI). The normalization was conducted to compare correlation coefficients between RT and M-PUI without contamination by the variation differences in RT and M-PUI among individuals (and among different sizes of smoothing, in the case of M-PUI). The correlation coefficients were calculated with M-PUI and RT normalized within individuals (and within different sizes of smoothing, in the case of M-PUI). We logarithmically transferred the RT, subtracted it by the mean RT, and divided it by the standard deviation of the RT within each participant (i.e., we obtained z-scores with zero mean and one standard deviation). The identical procedure was followed to normalize the M-PUI within each size of the smoothing window size. It is not statistically appropriate to calculate correlation coefficients by including independent and non-independent data points (i.e., some data points obtained from one individual and others obtained from different individuals). Therefore, the correlation coefficients were first calculated within each individual. Then, we examined the mean correlation coefficients between M-PUI and RT among individuals and the statistical significance of correlation coefficients per participant. Fisher’s z-transformation was performed on Pearson’s correlation coefficient r, then back-transformed to the r-value after the mean z was calculated to elucidate the mean correlation coefficients following the recommended statistical procedures [23]. Nonparametric permutation tests were used to determine whether the correlation coefficients were statistically significant. We expected a positive correlation between M-PUI and RT if the M-PUI reflected fluctuating levels of vigilance per trial.

Finally, we conducted two analyses that provide significant insights from both practical and theoretical perspectives. We expected that one analysis would indicate whether RTs would also be reflected in M-PUI as measured by other eye trackers with low temporal resolution and would also evaluate the effectiveness of M-PUI for different temporal resolutions in a way different from the previous correlation coefficient analysis (the panel in the middle row of Fig 4). For this purpose, we down-sampled the pupilar diameter’s time-series before the M-PUI calculation and then examined the mean correlation coefficients between intra-individually normalized RTs and intra-individually normalized M-PUI calculated from the down-sampled time-series of pupilar diameter. Down-sampling was done on the time-series of pupilar diameters at a ratio of 1/10 (from 1,000 Hz to 100 Hz), 1/20 (to 50 Hz), and 1/40 (to 25 Hz). Hanning windows of 5, 10, and 20 points were used to smooth the data. These correspond to windows sizes of 50, 100, and 200 ms in the down-sampled case of 100 Hz, 100, 200, and 400 ms in the down-sampled case of 50 Hz, and 200, 400, and 800 ms in the down-sampled case of 25 Hz. We conducted the identical normalization as above within each individual for each down-sampled data. If M-PUI reflected short-term vigilance levels robustly, we expected to find positive correlation coefficients even in the down-sampled data. In addition, if the effective time resolution was robust, the M-PUI from the size of the window that produced a large effect size in the previous correlation coefficient analysis should also have a significant effect size in this analysis.

Another analysis examined the time range relative to the target onset to estimate short-term vigilance (the panel in the bottom row of Fig 4). This indicates how many seconds the effect of M-PUI lasts, which is important from both practical and theoretical perspectives. This analysis compared the mean correlation coefficients between intra-individually normalized RT and intra-individually normalized M-PUI calculated at different time points using a Hanning window size of 50 ms. M-PUIs were calculated from eight types of pupilar time-series: from 1,000 ms before the target presentation to the target presentation (including all the trials), from 2,000 ms before the target presentation to 1,000 ms before the target presentation (including trials with a waiting time above 2,000 ms), from -3,000 ms to -2,000 ms (including trials above 3,000 ms), from -4,000 ms to -3,000 ms (including trials above 4,000 ms), from -5,000 ms to -4,000 ms (including trials above 5,000 ms), from -6,000 ms to -5,000 ms (including trials above 6,000 ms), from -7,000 ms to -6,000 ms (including trials above 7,000 ms), and from -8,000 ms to -7,000 ms (including trials of 8,000 ms). We conducted these identical normalizations within each individual for each time point. If M-PUI reflected real-time vigilance levels, then we expected that the larger the correlation coefficient, the closer the M-PUI calculation would be to the target’s point of appearance.

### Results

**Fig 5:**
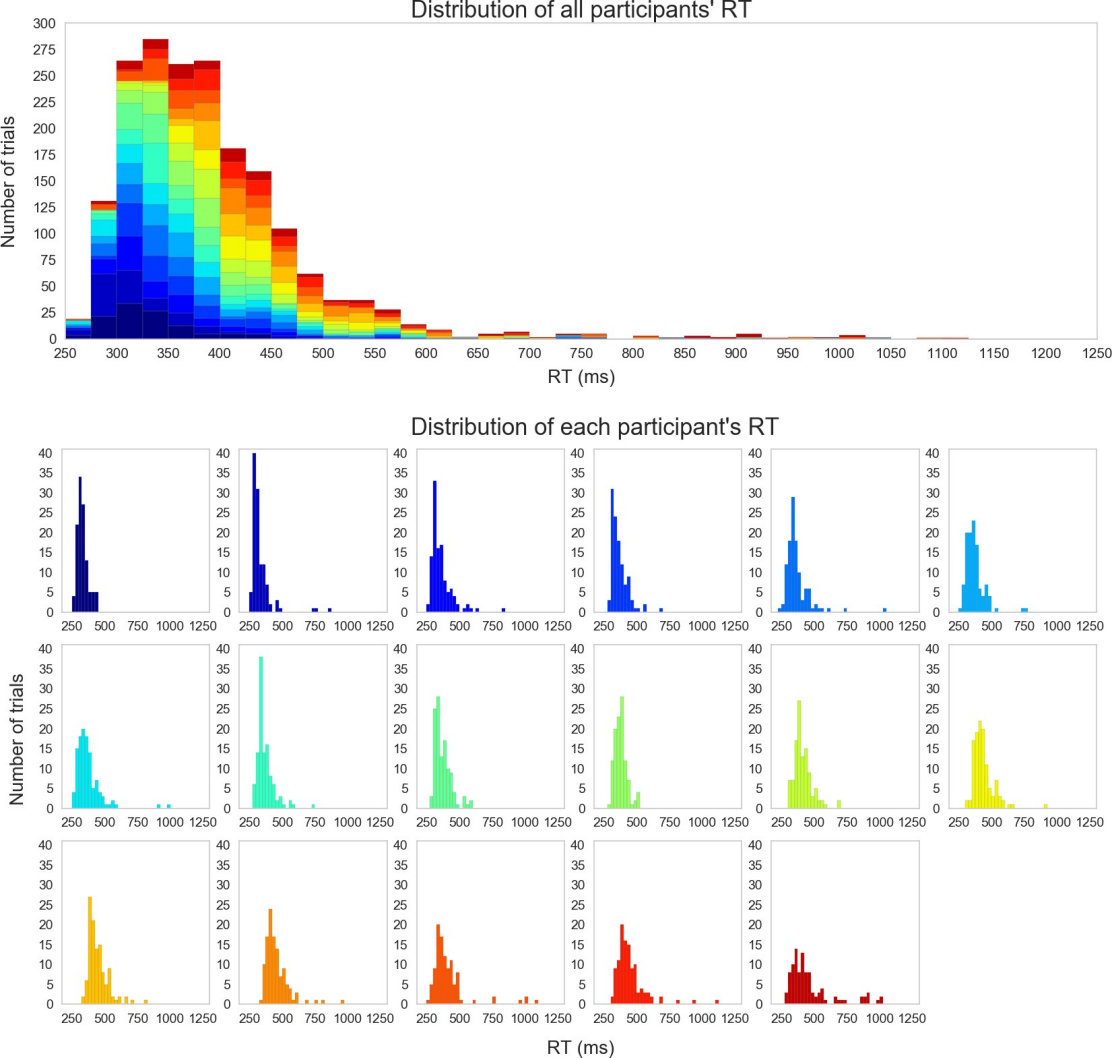
Distributions of RTs on all valid trials. The upper panel shows a histogram of the RT distributions of all participants. The lower panel shows RT distributions of individual participants, starting with the individual with the shortest mean RT. The different colors represent histograms of different individuals, which are common to the upper and lower figures. Note that two trials (4,988 ms and 1,662 ms, respectively) were not shown for visual purposes.

Nineteen participants provided valid pupilar data after excluding one participant due to technical errors. Two participants that had their eyelids closed during one part of the experiment were excluded from the analysis. The total number of trials was 1,972. The participants responded correctly to most trials, with no miss trials, only nine false alarm trials, which included anticipatory reactions with RTs of 150 ms or less, and 52 invalid trials, in which the eyes were entirely closed. Thus we excluded from the analysis 61 of 1,972 trials. The average number of valid trials for each participant was 112.41 (*SD* = 4.10). The distributions of RTs on all valid trials are shown in Fig 5.

We compared the mean M-PUI calculated from trials with long RT) and short RT (the top left and top middle panels of Fig 4).

First, in the case of the fixed thresholds of 500, 450, and 400 ms (the top left panel of Fig 4), paired-samples t-tests revealed that, for all the thresholds, mean M-PUIs were larger for long RTs than for short RTs for Hanning window sizes from 20 ms to 140 ms, with the largest effect sizes for 70 or 80 ms windows (corrected significance level of *p* < .0022). A summary of these statistics is provided in the upper part of Table 1.

**Table 1:** Summary of statistics testing differences between mean M-PUIs calculated from time-series for all smoothed pupilar diameters for long and short RTs.

Threshold		Mean M-PUI			Mean RT (ms)	Mean number of trials
		Lower limit case	Maximum effect case	Upper limit case		
Fixed at 500 ms	Long RT	0.67 ± 0.26	0.49 ± 0.21	0.44 ± 0.20	665.27 ± 177.62	10.53 ± 8.33 (Minimum = 3)
	Short RT	0.55 ± 0.20	0.35 ± 0.11	0.31 ± 0.10	374.69 ± 27.67	101.88 ± 9.90 (Minimum = 75)
	Smoothing window	20 ms (*t*(15) = 3.51, *d* = 0.50)	70 ms (*t*(15) = 3.66, *d* = 0.79)	180 ms (*t*(15) = 3.37, *d* = 0.76)	―	―
Fixed at 450 ms	Long RT	0.69 ± 0.28	0.50 ± 0.22	0.47 ± 0.22	566.11 ± 79.79	20.00 ± 14.19 (Minimum = 6)
	Short RT	0.54 ± 0.20	0.33 ± 0.11	0.31 ± 0.10	374.69 ± 27.67	92.41 ± 15.09 (Minimum = 64)
	Smoothing window	20 ms (*t*(15) = 3.52, *d* = 0.61)	80 ms (*t*(15) = 3.45, *d* = 0.90)	140 ms (*t*(15) = 3.36, *d* = 0.88)	―	―
Fixed at 400 ms	Long RT	0.64 ± 0.24	0.45 ± 0.18	0.40 ± 0.17	495.19 ± 50.86	39.76 ± 24.49 (Minimum = 8)
	ShortRT	0.52 ± 0.18	0.32 ± 0.09	0.28 ± 0.08	347.89 ± 18.92	72.65 ± 24.91 (Minimum = 29)
	Smoothing window	20 ms (*t*(16) = 4.19, *d* = 0.54)	70 ms (*t*(16) = 3.83, *d* = 0.88)	200 ms (*t*(16) = 3.38, *d* = 0.82)	―	―
Adjusted at 90th	Long RT	0.70 ± 0.28	0.51 ± 0.23	0.40 ± 0.19	627.30 ± 205.54	11.41 ± 0.84
	Short RT	0.54 ± 0.20	0.33 ± 0.11	0.27 ± 0.09	372.47 ± 38.05	101.00 ± 3.56
	Smoothing window	20 ms (*t*(16) = 3.83, *d* = 0.66)	90 ms (*t*(16) = 3.90, *d* = 0.94)	400 ms (*t*(16) = 3.33, *d* = 0.83)	―	―
Adjusted at 60th	Long RT	0.60 ± 0.23	0.41 ± 0.16	0.37 ± 0.14	475.77 ± 74.49	44.29 ± 1.67
	Short RT	0.53 ± 0.20	0.31 ± 0.11	0.29 ± 0.10	346.42 ± 33.21	68.12 ± 2.68
	Smoothing window	20 ms (*t*(16) = 3.88, *d* = 0.35)	90 ms (*t*(16) = 3.33, *d* = 0.66)	180 ms (*t*(16) = 3.33, *d* = 0.66)	―	―
Adjusted at 30th	Long RT	0.58 ± 0.21	0.36 ± 0.13	0.23 ± 0.07	429.52 ± 54.30	77.47 ± 3.27
	Short RT	0.51 ± 0.19	0.28 ± 0.09	0.19 ± 0.06	325.97 ± 30.63	34.94 ± 2.94
	Smoothing window	20 ms (*t*(16) = 4.67, *d* = 0.32)	120 ms (*t*(16) = 4.67, *d* = 0.71)	1,000 ms (*t*(16) = 3.53, *d* = 0.60)	―	―

Next, we conducted paired-samples t-tests for individually adjusted thresholds of 90, 60, 30 percentile points (the top middle panel of Fig 4), revealed that mean M-PUIs of all thretholds were larger for long RTs than for short RTs for 20 ms to 180 ms Hanning windows (*p* < .0022). The largest effect was observed for 90 to 120 ms windows. A summary of these statistics is provided in the lower part of Table 1.

The "Threshold" column shows RT thresholds dividing the trials. The "Mean M-PUI" column shows the mean M-PUIs for cases the "Lower limit," "Maximum effect," and "Upper limit" of the smoothing window size used in calculating M-PUIs. The "Lower limit " represents the smallest window size with a significant difference in mean M-PUIs, and the "Maximum effect" represents the window size for the largest effect size. The "Upper limit " represents the largest window size with a significant difference in mean M-PUIs. The "Mean RT" and the "Mean number of trials" columns separately show the mean RTs across participants and the mean number of trials per participant. The "Long RT" and "Short RT" rows separately list the statistics in trials with long and short RTs. The "Smoothing window" row lists the specific window sizes for which the M-PUIs are calculated along with the results of statistical tests. The plus-minus signs represent standard deviations. One participant was excluded from the fixed 500 and 450 ms threshold for the lack of long RTs (>450 ms).

**Fig 6:**
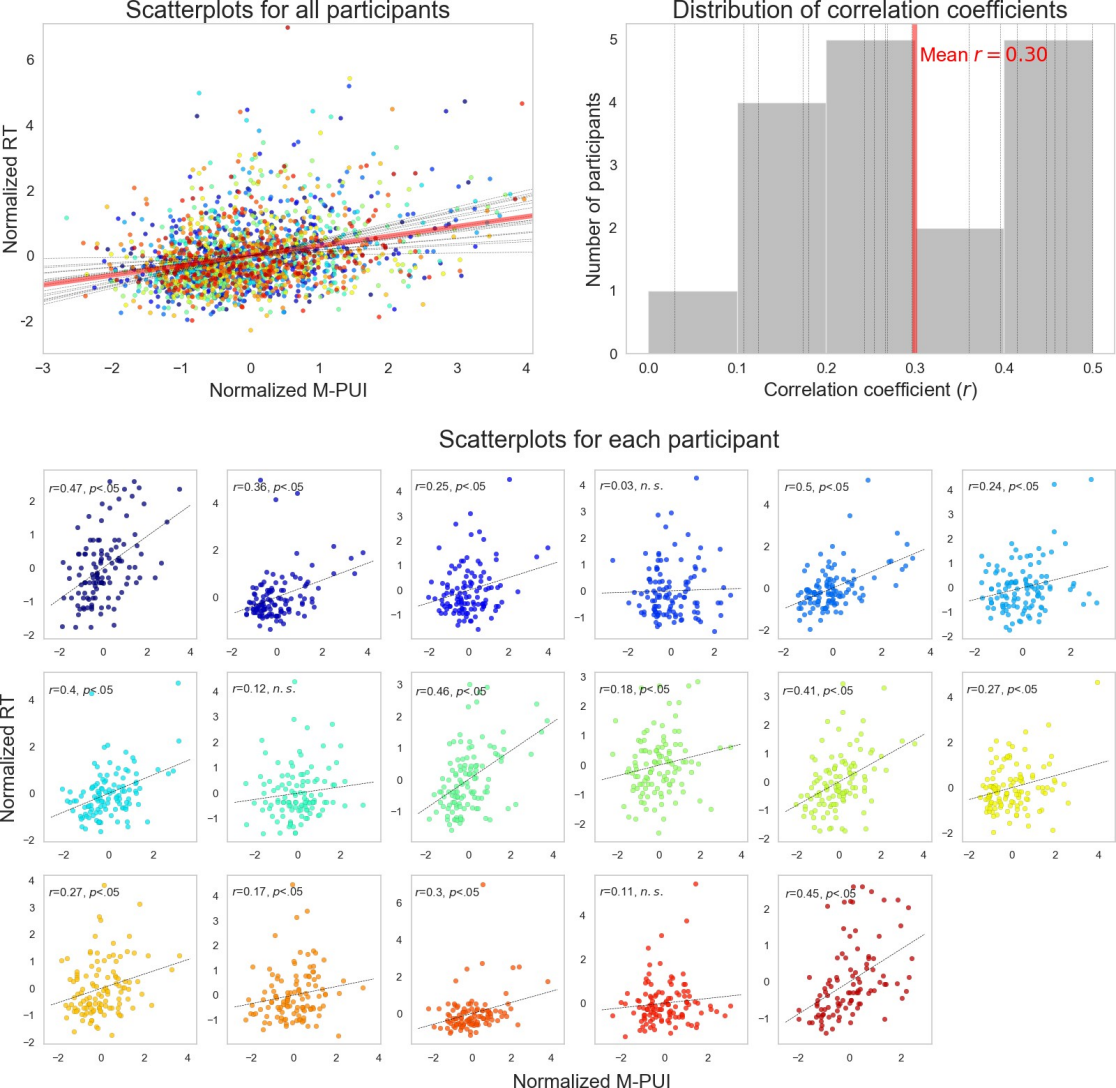
Scatterplot and distributions of each participants’ and correlation coefficients across all participants between normalized RT and normalized M-PUI for the 50 ms Hanning window. The top left panel represents the scatterplot between normalized RT and M-PUI for the 50 ms Hanning window for each participant and all participants. The top right panel represents the distributions of correlation coefficients between normalized RT and normalized M-PUI for the 50 ms Hanning window for each participant and all participants. The bottom panel represents all scatterplots for each participant. The different colors represent scatterplots of different individuals that are common to Fig 5. The solid red line in these panels shows the mean magnitude of the correlation coefficient for all participants, and the dotted black lines show the magnitudes of the correlation coefficient for each participant.

In the most theoretical test, we examined the correlation coefficients (Pearson’s r) between intra-individually normalized M-PUI and intra-individually normalized RT for all trials (the top right panel of Fig 4). The scatterplot and distributions of correlation coefficients between the normalized RT and normalized M-PUI are shown in Fig 6 for the 50 ms window. Although there are variations among participants, there is a shared positive correlation coefficient with a mean of 0.30 between M-PUI and RT within each participant.

**Fig 7:**
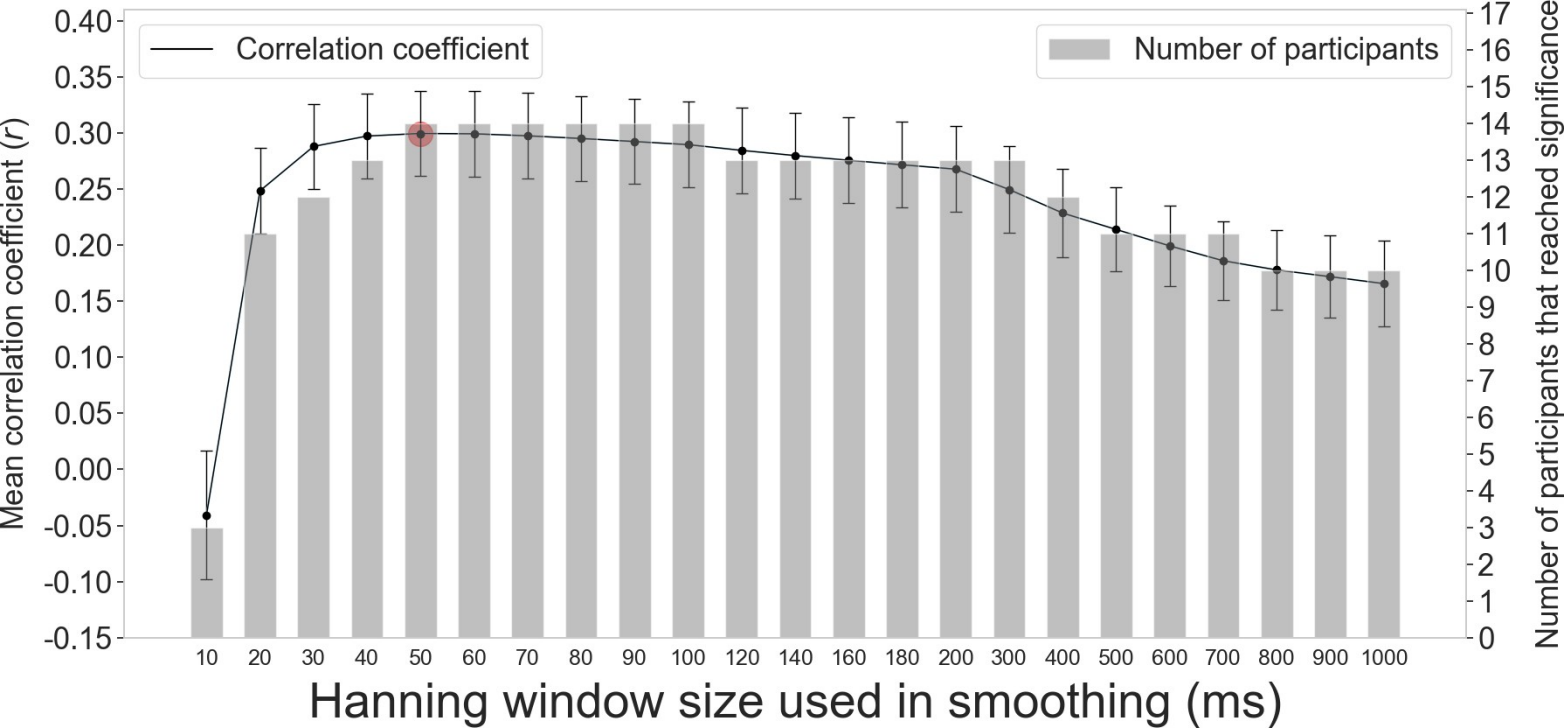
Mean correlation coefficients between normalized M-PUI and normalized RT for each Hanning window size and numbers of participants whose correlation coefficient reached significance. Red dots indicate the points where detailed scatterplots are shown in Fig 6. Error bars represent standard errors.

Fig 7 shows the mean correlation coefficients (Pearson’s r) among participants for each size of the Hanning window and the numbers of participants whose correlation coefficients reached a significance level of *p* < .05. All the mean correlation coefficients were a positive, except the 10 ms Hanning window (possibly caused by measurement noise). The mean correlation coefficients were slightly larger for windows between 30 and 200 ms than those outside this interval, and the number of participants that reached significance was largest for windows between 50 and 100 ms. As indicated in Figs 6 and 7, the largest mean correlation coefficient was for the 50 ms window (mean *r* = 0.30, *SEM* = 0.15), with the largest number of participants reached significance (*N* = 14). All the data points in the correlation coefficient calculation must be independent. Therefore, we do not consider the correlation coefficients between normalized M-PUI and RT calculated from all data points as formal. However, the results, in this case, were nearly identical.

**Fig 8:**
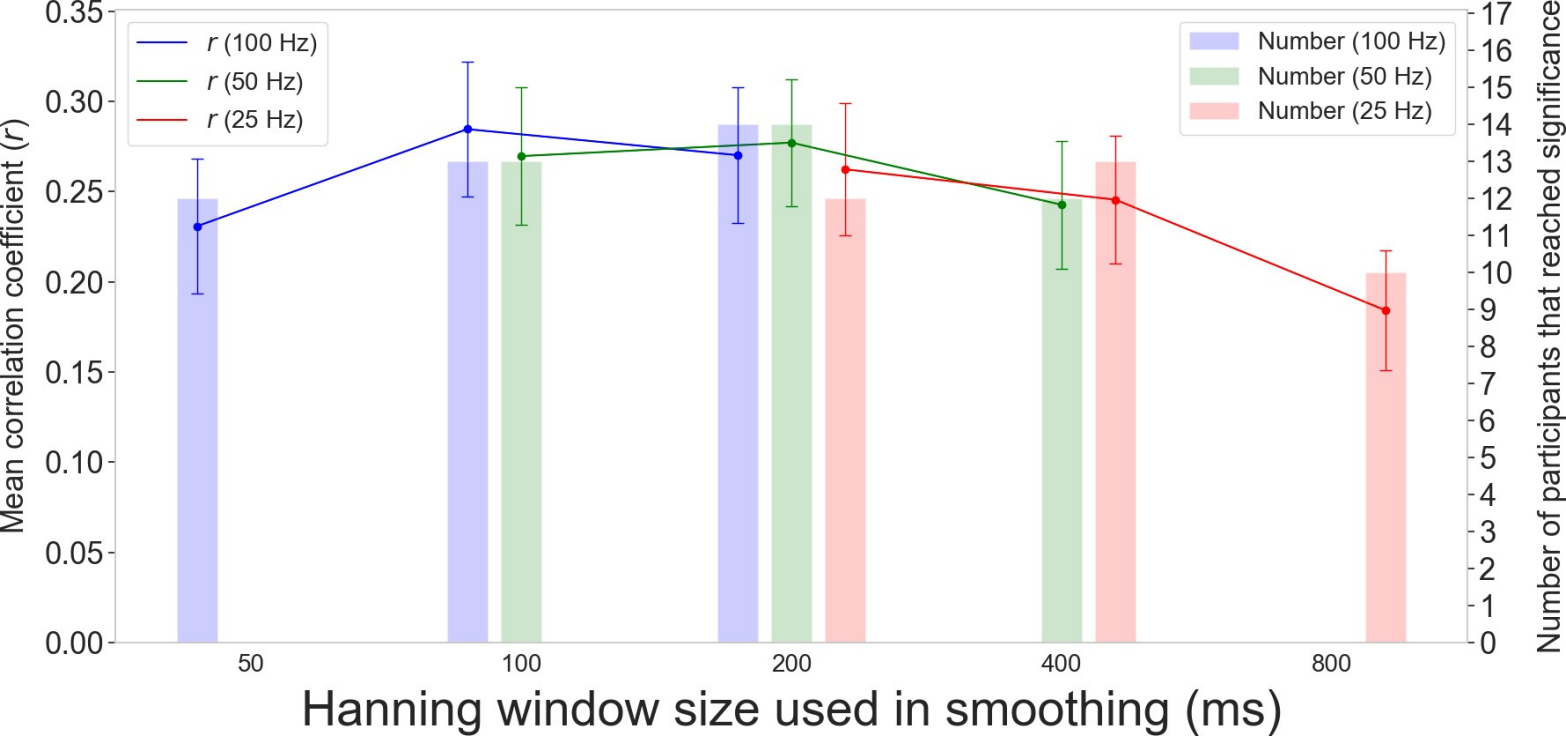
Mean correlation coefficients between normalized M-PUI and normalized RT for all down-sampled time-series of pupilar diameters and numbers of participants whose correlation coefficients reached significance. The blue line represents the mean correlation coefficients between normalized RT and normalized M-PUI calculated from the pupilar diameters down-sampled to 100 Hz. The blue bars represent the numbers of participants that reached significance in the case of 100 Hz. The green line represents the mean correlation coefficients between normalized RT and normalized M-PUI calculated from the pupilar diameters down-sampled to 50 Hz, and the green bars represent the numbers of participants that reached significance in the case of 50 Hz. The red line represents the mean correlation coefficients between normalized RT and normalized M-PUI calculated from the pupilar diameters down-sampled to 25 Hz, and the red bars represent the numbers of participants that reached significance in the case of 25 Hz.

Finally, we conducted two analyses that provide important insights from practical and theoretical perspectives (the panels in the middle and bottom row of Fig 4). We examined the correlation coefficients between intra-individually normalized RT and intra-individually normalized M-PUI calculated from the down-sampled time-series of pupilar diameters (Fig 8). Positive correlations between normalized M-PUI and normalized RT were found for all Hanning windows, although mean correlation coefficients were slightly larger for windows between 100 and 200 ms than those outside this interval. The largest correlation was for the 100 ms window (mean *r* = 0.28, *SEM* = 0.15). Most participants reached significance for the 100 ms window (*N* = 13, for 100 and 50 Hz), while the largest number of participants reached significance for the 200 ms window (*N* = 14, for 100 and 50 Hz).

**Fig 9:**
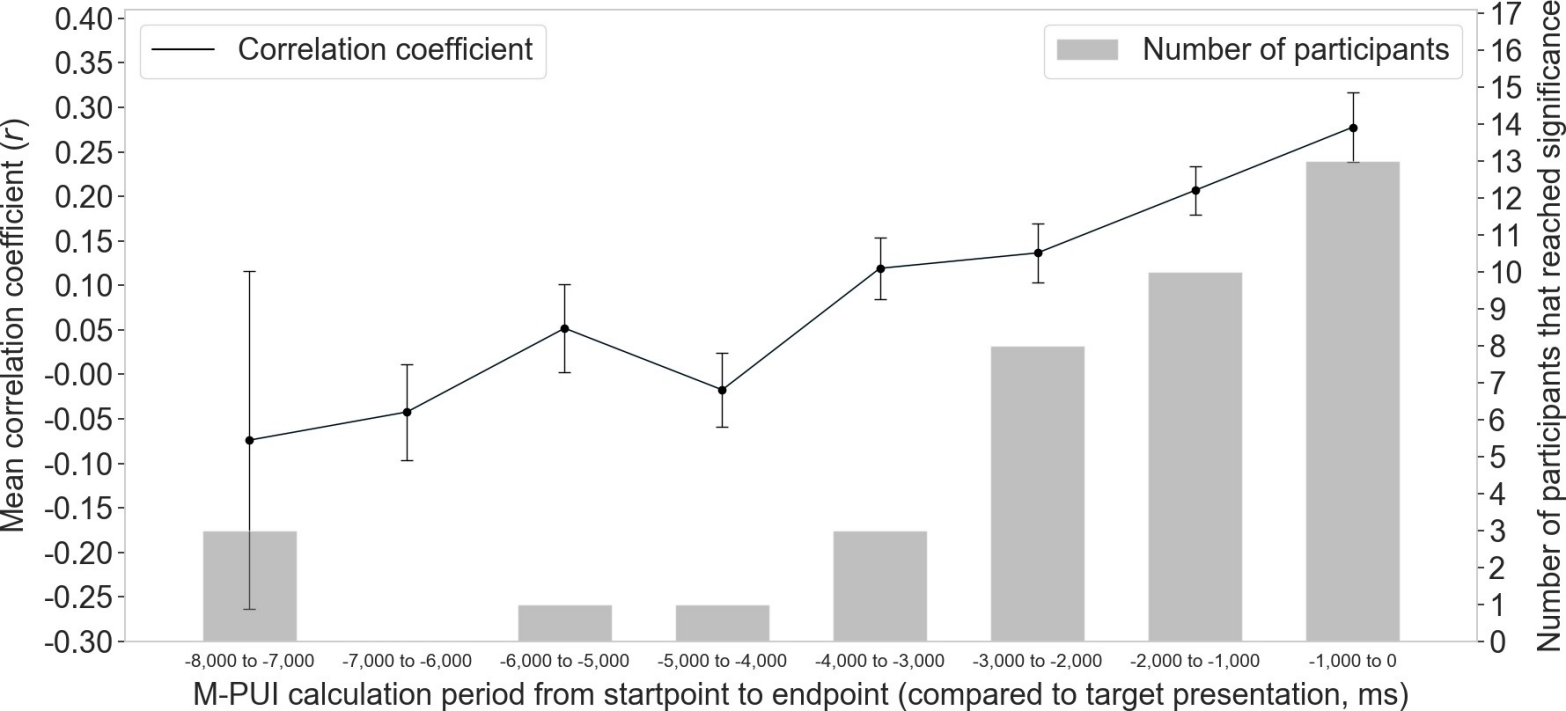
Mean correlation coefficients between normalized M-PUI and normalized RTs for different time points and numbers of participants whose correlation coefficients reached significance.

Further, we compared the mean correlation coefficients between intra-individually normalized RT and intra-individually normalized M-PUI for the Hanning window of 50 ms, calculated from different time points (the panel in the bottom row of Fig 4). The results (Fig 9) indicated that the normalized M-PUIs calculated from time intervals within 1,000 ms before the target presentation had the largest positive correlation (*r* = 0.27, *SEM* = 0.15) with normalized RT, with the largest number of participants that reached significance (*N* = 13). The correlation coefficient decreased as the time intervals moved away from the time of target presentation, with the number of participants that reached significance also decreasing. Only when the interval was less than 2000 ms (from -2,000 ms to -1,000 ms, and from -1,000 ms to 0 ms) was the number of participants that reached significance greater than half of the total number of participants.

## Discussion

### Validity of M-PUI for estimating real-time vigilance: Practical guidelines

The analysis results from a practical perspective suggest that M-PUI can be used to estimate and predict a fluctuating level of vigilance. Evidence supporting this conclusion can be seen in comparing M-PUIs between short/long RTs using a fixed threshold, the effectiveness of M-PUIs calculated from the down-sampled data, and the adequate time intervals of M-PUI (see Fig 4). The comparison of M-PUIs between short/long RTs based on a fixed threshold showed that the Hanning windows of 20 to 140 ms gave the most valid results. We can imagine a practical situation of a widely-used system that does not adapt to individuals, such that the performance decrement threshold at which errors occur is the same for everyone; in this system, human error is mitigated through the detection of a decrease in a short-term vigilance level beyond this threshold. The effect size of M-PUI was large enough to be statistically significant even in such a practical, real-world situation in which individual differences cannot be considered or controlled. It is essential to point out that many previous studies have used the number of trials that exceed a fixed threshold (500 ms, especially) as an indicator of decreased long-term vigilance [12]. The M-PUI, which is thought to reflect the state of the psychophysiological mechanism that supports short-term vigilance, differed when the fixed threshold was used suggests that the fixed threshold also has practical validity in PVT analysis on a trial-by-trial basis.

Further, M-PUI calculated from the time-series of pupilar diameters measured with a low-time resolution eye tracker also reflected short-term vigilance levels when window sizes smoothed pupilar diameter’s characteristics up to approximately 200 ms. Note that the effectiveness of the M-PUI calculated from the small window (50 ms) was slightly reduced. This suggests that when the time resolution of the eye tracker is low and the number of samples (i.e., the number of pupilar diameter time-series) considered in smoothing is too small, the M-PUI calculation accuracy can be lower. Thus, when calculating M-PUI from pupilar diameters acquired at a low temporal resolution, smoothing at a medium resolution, such as 100 ms, is more appropriate than smoothing at a fine resolution, such as 50 ms.

Furthermore, only normalized M-PUIs calculated within 1,000 ms (or, at most, 2000 ms) before the target presentation was helpful in vigilance estimation. These findings identified valid time intervals for estimating real-time vigilance during real-world activities.

### Characteristics of M-PUI that reflect vigilance: Theoretical implications

The results of the analyses from a theoretical perspective suggest that there is a generalizable relationship between M-PUI and fluctuating levels of vigilance. Supporting evidence emerged from several sources: the comparison of M-PUIs between short/long RTs using individually adjusted thresholds, the correlations between the intra-individually normalized M-PUI and intra-individually normalized RT, calculations of M-PUI from the down-sampled data, and examination of the adequate time intervals of M-PUI (see Fig 4). When the short-term vigilance levels were divided using individually adjusted thresholds, M-PUIs differed for Hanning windows from 20 to 180 ms. When we normalized M-PUI and RT within each participant, intra-individually normalized M-PUIs for Hanning windows of 50 to 100ms were most positively correlated with intra-individually normalized RTs. These effective time resolutions did not change for the down-sampled data. Regarding the mechanism of the relationship between M-PUI and RT, it is essential to note that the effect size of M-PUI was most prominent when the size of the smoothing window was at a fine resolution smaller than those used in the original PUI calculation. Interestingly, the M-PUI no longer reflected the level of short-term vigilance when the time of calculation of the M-PUI was three seconds away from the short-term vigilance measurement (target presentation). The effectiveness of M-PUI on short timescales suggests that the M-PUI does not reflect states that change on long timescales, from tens of seconds to minutes.

### LC activity underlying pupillary fluctuations

The pupilar diameter is known to be strongly correlated with LC activity [13]. The LC affects attention through the noradrenaline pathway in two different activities [13, 14]. In the tonic activity, the LC is activated at approximately 0.1–5.0 Hz and adjusts the arousal, which affects the pupilar diameter. In the phasic activity, the LC is activated at approximately 10–20 Hz and promotes responsiveness to novel or task-relevant stimuli through the noradrenaline pathway, which is also reflected in phasic pupilar dilation.

The present results indicated that the amplitude of pupillary fluctuations assessed by time-series data smoothed by a fine resolution window of approximately 50–100 ms was best correlated with the degree of short-term vigilance. In other words, the frequency of pupillary fluctuations with an amplitude that best correlated with the degree of short-term vigilance had a temporal resolution of approximately 10–20 Hz (1000/100 ms to 1000/50 ms). 10–20 Hz is different from the frequency range of spontaneous pupillary fluctuations typically associated with sleepiness because sleepiness waves occur below 0.5–0.8Hz [11, 24].

Typically, sleepiness-related long-term pupillary fluctuations are interpreted as reflecting the ANS state. Fluctuations between sympathetic and parasympathetic control might occur during periods of reduced long-term vigilance reflected in large pupilar diameter fluctuations [9, 11]. One study suggests that the ANS state is mediated by the Edinger-Westphal nucleus in the midbrain [9] controlled by the LC [10]. Therefore, sleepiness-related slow pupillary fluctuations below 0.5–0.8 Hz identified in previous studies might reflect the LC’s tonic activity (0.1–5.0 Hz). Indeed, the adaptive changes that reduce the LC’s tonic arousal level are considered to occur when there is inadequate stimulation from the environment, which correlates with very slow pupillary fluctuations [5]. Participants being forced to wait in the dark for an extended time for PUI measurement is just such a situation when LC’s tonic activity decreases. However, the LC’s tonic activity might sometimes increase as participants try to resist sleeping and remain awake because of the requirement to keep their eyes open during PUI measurement. These conflicts appear more strongly at lower levels of arousal, such as in sleep-deprived people. Therefore, it is likely that larger pupillary fluctuation amplitudes occur at a very slow timescale.

On the other hand, short-term pupillary fluctuations measured with a temporal resolution of approximately 10–20 Hz, with an amplitude that correlated with the short-term vigilance level in each trial of this study, might be related to the LC’s phasic activity that occurs at 10–20 Hz. As mentioned earlier, the LC’s phasic activity plays an essential role in NE release, which enhances the novel or task-relevant stimuli critical for the current task [13, 14]. The LC’s phasic activity is reciprocally innervated by the anterior cingulate cortex (ACC) [13], which is a component of the salience network that is involved in orientation to salient stimuli [25]. Also, task-relevant stimuli’s enhancement by LC’s phasic activity has been observed when the target appears in space or at a time indicated by a spatial (e.g., orienting effect [26–28]) or a timing cue [20]. Then, the phasic dilation in pupilar diameter, which might be due to the release of NE, was more robust, and the response to the target was faster than when the target appeared without the cue. It has been suggested that the LC’s phasic activity enhances target detection with task relevance weighted by something such as a cue. Considering the frequency band match, the phasic enhancement of target detection might be one mechanism of the correlation between short-term fluctuations of pupillary amplitudes and short-term vigilance levels.

A discussion of the relationship between M-PUI and short-term vigilance levels is incomplete without describing the concept of neuron coupling in the LC. Usher, Cohen, Servan-Schreiber, Rajkowski, and Aston-Jones [15] suggested that neurons in the LC are coupled (synchronized) when they are highly responsive to task-relevant stimuli, and the task performance is optimal. In contrast, they are decoupled (desynchronized) when the responsiveness to task-relevant stimuli is low and the task performance is suboptimal. These current results can be explained based on this model. The correlation between short-term pupillary fluctuation amplitude and short-term vigilance level did not decrease considerably as the smoothing window size increased to approximately 200 ms. That is, short-term pupillary fluctuations with amplitudes correlating with short-term vigilance levels, as observed in the present study, might not be involved in synchronized neural activity at a specific frequency, such as alpha waves in the case of attention [29]. Instead, the reduced vigilance was possibly associated with the state of momentarily higher or lower overall LC activity over a wide frequency range. A possible mechanism underlying this phenomenon is elevated randomness, or desynchronization, which causes the LC’s neurons to transiently change their firing rate over a wide range of frequencies, thereby accidentally increasing or decreasing the overall LC activity in different frequency bands. Such randomness is also associated with the LC’s phasic activity. A model of the LC’s phasic activity proposes that the LC’s neurons become uncorrelated and randomized relative to each other when the motivation to stop the task increases, thereby reducing the responsiveness to task-relevant stimuli [13, 30]. This suggests that when the motivation for the current task is high and the LC’s randomness is low, the LC’s phasic activity differs significantly between intervals with and without task-relevant stimuli, resulting in phasic enhancement of only task-relevant stimuli. On the other hand, when randomness reflecting a tendency to move away from a task is observed in the LC, there is no difference in LC’s phasic activity between intervals with and without task-relevant stimuli, which results in exploring novel environments rather than enhancing task-relevant stimuli. Therefore, it is possible that the characteristics of short-term pupillary fluctuations, including the M-PUI, might facilitate estimating engaging with a current task through the enhancement of task-relevant stimuli. Further research using tasks measuring the orienting component of attention involved in phasic enhancement of task-relevant stimuli, including the Attention Network Test [26, 27, 28] (i.e., spatial enhancement) or the Rapid Serial Visual Presentation Task [31–33] (i.e., temporal enhancement) would be needed further to elucidate the relationship between M-PUI and task performance.

### Limitations and future directions

Experimental conditions possibly affected the RT under short-term vigilance levels (RTs per trial) on a trial-by-trial basis. A crucial experimental condition in PVT is the target presentation’s variable schedule (i.e., different waiting times between the start of a trial and the target presentation). For example, it is known that RT increases as the timing of the target presentation is moved closer to the start of a trial in tasks with different waiting time lengths (i.e., "foreperiod effect") because participants develop adaptations to the experimental situation such that the probability of a target appearance is estimated low immediately after starting a trial [34]. Its probability is estimated to increase as the time without a target presentation during a trial increases. Besides, this trend in RT is enhanced by the previous trial’s waiting time difference (i.e., "sequential effect"). These effects have been recently reported to affect short-term vigilance levels (i.e., RTs per trial) in PVT [35] and to be affected by long-term vigilance levels [36]. Therefore, we speculated that these effects might be included in our definition of short-term vigilance in PVT. The concept of LC’s phasic activity (e.g., the temporal enhancement of the task-relevant stimuli) might be related to participants’ adaptation, increasing the responsiveness to targets at specific timings when the probability of target appearance is high. Therefore, the relationship between M-PUIs and RTs could be partially mediated by increased responsiveness as the time without a target presentation increases. There is one important point to consider in this regard. We verified that correlations between M-PUI and RT were, at least, not sorely dependent on the current trial’s waiting time. Specifically, we observed positive correlations between M-PUI and RT, normalized within each waiting time rather than within each participant. In other words, it was confirmed that the relationship between M-PUI and RT remains even after controlling the tendency to increase responsiveness to a target during the waiting time of each PVT trial (i.e., the foreperiod effect) in our study. Rather, the trial-by-trial fluctuations in the increase in responsiveness during the waiting time (i.e., the trial-by-trial differences in the foreperiod effect) might be a source of fluctuations in the short-term vigilance levels. Such trial-by-trial fluctuations might include the sequential effect (see the arousal-related higher-order sequential effect [37]). Future studies might show that some M-PUI fluctuations’ effects in short-term PVT vigilance levels can be explained by factors related to experimental conditions such as the previous trial’s waiting time and could also help elucidate the underlying mechanism of short-term vigilance level.

We did not investigate long-term vigilance levels because of the scope of this study. However, since long-term vigilance level (i.e., overall RT in one PVT session) is calculated based on the sum of short-term vigilance level (i.e., RT per trial), clarifying the relationship between them is crucial for elucidating the nature of vigilance tasks that are traditionally considered as long-term. One possible approach in this direction is to examine the modulation of short-term vigilance by long-term vigilance, which could be determined by examining whether variations in long-term vigilance characteristics are reflected in psychophysiological indicators of variations in short-term vigilance such as M-PUI. For example, distributional analysis of RTs within each PVT session might be used for M-PUI [38, 39]. The distributional analysis can reveal whether the increase in overall RT (e.g., mean RT) in one PVT session, along with the long-term vigilance decrement, might be due to a generic slow-down of all responses or a selective slow-down of specific responses within RT distributions’ long percentiles [39]. It has been reported that the increase in overall RT (i.e., mean RT) when long-term vigilance levels are reduced by extended continuous wakefulness (i.e., sleep deprivation) is due to a selective slow-down of responses within long percentiles [39]. Therefore, we can draw the distributions of M-PUI correlating with RTs per trial by manipulating long-term vigilance through sleep deprivation and observe whether M-PUI within long percentiles and RT selectively increases. A selective increase in the distribution of the M-PUI might suggest that the M-PUI reflects short-term vigilance level, which is modulated by the long-term vigilance levels. The LC’s phasic activity [13], which might be related to the M-PUI degree associated with RT per trial is modulated by the tonic LC activity [5, 13] related to the degree of PUI associated with overall RT within each session [12]. Therefore, this relationship could be suggestive of short-term vigilance modulation by long-term vigilance. Interestingly, comparing M-PUIs between trials divided by thresholds at the 90th percentile point in our data resulted in a relatively higher M-PUI effect and a higher mean RT than the 60th and 30th percentile thresholds. Since relative sleep deprivation is reported to be expected even in healthy populations [5], this selective increase in M-PUI and RT within long percentiles might reflect sleepiness among the study’s participants. However, separating the experimental participants into groups according to the time of day (11:00–17:00) revealed neither a significant sleepiness-related trend in RT nor M-PUI nor a relationship between RT and M-PUI. Therefore, further studies with strict experimental controls for sleep-related factors are warranted. The M-PUI does not entirely reflect long-term vigilance levels because the temporal resolution of the M-PUI effect is different from long-term effects. Previous studies that examined pupil sizes’ correlations with short-term vigilance have entirely relied on separate comparisons between the shortest and the longest RTs within short and long percentile points [20]. Therefore, their distributional characteristics remain to be clarified by future research.

Several remaining issues warrant further investigation. Because this study was generally conducted in a dark environment, it remains unclear whether the same results can be obtained in different brightness environments. Some studies have found that the pupilar diameter may differ in dark and light environments [24]. Thus it is necessary to investigate the effectiveness of M-PUI in different brightness conditions. Further, although the M-PUI reflected the fluctuating level of vigilance in most individuals, the extent of its effectiveness varied among individuals. For example, no significant correlations were obtained for three experimental participants. Such results may be clarified by analyzing the relationship between M-PUI effect size and individual differences including cognitive failure liability [38] or subjective states such as task engagement, distress, and worry [40]. These future studies should have important implications from both practical and theoretical perspectives.

## Conclusion

M-PUI can be used to estimate and predict short-term vigilance levels in a PVT, even without controlling individual differences in practice. Theoretically, this estimation is optimal for time-series of pupilar diameters within one or two seconds before target onset after smoothing by Hanning windows of 50 to 100 ms. This method is expected to effectively assess even low temporal resolutions (e.g., 50 Hz). It is expected that future investigations of short-term pupillary fluctuations would further contribute to elucidating the psychophysiological mechanisms of short-term vigilance.
